# Redistribution of CB1 Cannabinoid Receptors in the Acute and Chronic Phases of Pilocarpine-Induced Epilepsy

**DOI:** 10.1371/journal.pone.0027196

**Published:** 2011-11-04

**Authors:** Mária R. Karlócai, Kinga Tóth, Masahiko Watanabe, Catherine Ledent, Gábor Juhász, Tamás F. Freund, Zsófia Maglóczky

**Affiliations:** 1 Institute of Experimental Medicine, Hungarian Academy of Sciences, Budapest, Hungary; 2 Department of Anatomy, Hokkaido University School of Medicine, Sapporo, Japan; 3 IRIBHM, Université Libre de Bruxelles, Brussels, Belgium; 4 Research Group of Proteomics, Institute of Biology, Eötvös Loránd University, Budapest, Hungary; Centre national de la recherche scientifique, University of Bordeaux, France

## Abstract

The endocannabinoid system plays a central role in retrograde synaptic communication and may control the spread of activity in an epileptic network. Using the pilocarpine model of temporal lobe epilepsy we examined the expression pattern of the Type 1 cannabinoid receptor (CB1-R) in the hippocampi of CD1 mice at survival times of 2 hours, 1 day, 3 days and 2 months (acute, latent and chronic phases). Based on the behavioral signs of the acute seizures, animals were classified as “weakly” or “strongly” epileptic using the modified Racine scale. Mice of the weak group had mild seizures, whereas seizures in the strong group were frequent with intense motor symptoms and the majority of these animals developed sclerosis in the chronic phase. In control samples the most intense staining of CB1-R-positive fibers was found in the molecular layer of the dentate gyrus and in str. pyramidale of the cornu Ammonis. In weak animals no significant changes were seen at any survival time compared to controls. In strong animals, however, in the acute phase, a massive reduction in CB1-R-stained terminals occurred in the hippocampus. In the latent phase CB1-R immunoreactivity gradually recovered. In the chronic phase, CB1-immunostaining in sclerotic samples was stronger throughout the hippocampus. Quantitative electron microscopic analysis showed an increase in the number of CB1-R-positive terminals in the dentate gyrus. Moreover, the number of immunogold particles significantly increased in GABAergic terminals. Our results suggest a proconvulsive downregulation of CB1 receptors in the acute phase most probably due to receptor internalization, followed by compensatory upregulation and sprouting in the chronic phase of epilepsy. In conclusion, the changes in CB1 receptor expression pattern revealed in this study are associated with the severity of hippocampal injury initiated by acute seizures that ultimately leads to sclerosis in the vulnerable regions in the chronic phase.

## Introduction

Epilepsy is one of the most common neurological diseases affecting 1–3% of the population [1], [2]. At the neuronal network level it manifests as states of pathological hyperexcitability and hypersynchronous activity. Imbalanced synaptic input may cause excessive neuronal activity, eventually leading to neuronal death and synaptic reorganization [3], [4], [5]. Understanding the underlying mechanisms is crucial for the development of new effective therapies.

The endocannabinoid system is involved in numerous physiological functions like food intake, pain sensation and memory formation. In the brain, the endocannabinoid system is responsible for retrograde synaptic signaling via CB1-R (Type 1 cannabinoid receptor) [6], [7], [8]. Endocannabinoids are released from the postsynaptic neurons in an activity-dependent manner, and bind to presynaptic CB1-Rs, thereby suppressing transmitter release from presynaptic terminals [9], [10], [11], [12], [13], [14], [15], [16], [17].

In addition to its physiological roles, this system was found to be affected in pathological processes as well. Controversial data were published regarding the effects of cannabinoids in epilepsy. On one hand, in an animal model of TLE (temporal lobe epilepsy), CB1-R agonists displayed anti-epileptic effects [18], in addition, CB1-Rs on glutamatergic axon terminals were shown to mediate anticonvulsant effect, by attenuating glutamate release [19],[20]. On the other hand, proconvulsive effects of CB1-R agonists were described as well [21], . Moreover, a CB1-R antagonist was shown to prevent the long-term increase in seizure susceptibility when applied in a certain time-window [24], [25].

Human studies showed that recurrent seizures may lead to an adverse reorganization of the endocannabinoid system and to the impairment of its protective effect [26], [27]. CB1-Rs located at inhibitory synapses can be upregulated in the dentate gyrus [28], whereas downregulation of CB1-Rs located at excitatory synapses may occur in the inner molecular layer of the dentate stratum moleculare [27]. In the pilocarpine model of epilepsy downregulation of CB1-Rs located at inhibitory synapses was found in the acute phase [29], [30], but upregulation occurred in the chronic phase [18].

However, a quantitative ultrastructural examination of CB1-Rs both at excitatory and inhibitory synapses in different phases of epileptogenesis has not been carried out in this model of epilepsy so far.

Using the pilocarpine model of temporal lobe epilepsy we examined the expression pattern of CB1-Rs on both glutamatergic and GABAergic axon terminals at light and electron microscopic levels in the hippocampi of CD1 mice. In the acute phase of epilepsy a considerable downregulation of the receptor was found. Effects of the loss of receptors were examined in mice lacking CB1-Rs. In the knock out animals we show that seizures are more severe in most animals, moreover, survival rates worsen during acute seizures compared to wild types. However, in wild type animals in the chronic phase an upregulation of CB1-Rs occurred throughout the hippocampus which may serve as a neuroprotective mechanism (via decreasing excitability and synchronization by reducing glutamate and GABA release). Nevertheless, during the acute seizures the high concentration of the ligand may be responsible for transient internalization/downregulation of the receptor [23], [31], [32] and increasing seizure susceptibility.

## Materials and Methods

### The model

Animals were kept under standard conditions with 12 h dark-light cycle; food and water were supplied ad libitum. Experiments were performed according to the guidelines of the Institutional Ethical Codex & the Hungarian Act of Animal Care & Experimentation (1998, XXVIII, Section 243/1998), The Animal Care and Experimentation Committee of the Institute of Experimental Medicine of Hungarian Academy of Sciences and the Animal Health and Food Control Station, Budapest, has approved the experimental design under the number of 2303/003/FŐV/2006. The experiments are in accordance with 86/609/EEC/2 Directives of European Community, which is in full agreement with the regulation of animal experiments in the European Union. All efforts were made to reduce the number of animals used and to minimize pain and suffering. For this animal model 20–30 g male CD1 mice (Harlan, Italy) and CB1-R knock-out CD1 mice were used [33]. Animals were assigned to control and experimental groups. Age-matched control mice were injected with physiological saline (12 animals, 0.1 ml/30 g) or scopolamine (12 animals 5 mg/kg). Since no difference was found between the two control groups, during the following experiments, control mice were injected with physiological saline. Experimental mice were injected with intraperitoneal Pilocarpine hydrochloride (340 mg/kg, Sigma) to induce status epilepticus (SE). Scopolamine methyl nitrate (5 mg/kg, Sigma) was injected 30 minutes in advance to prevent peripheral cholinergic effects of pilocarpine. No benzodiazepine treatment was used to stop seizures. By omitting antiepileptic drugs we were able to examine seizure-induced changes 2 hours post SE, without further alteration of the GABAergic function by additional drugs.

Animals were observed for two hours after the pilocarpine injection, and the behavioral signs of seizures were monitored and scored. At every 5 minutes or when behavioral changes occurred a score was determined. Acute seizures started 5–15 minutes after the pilocarpine administration (post pilo). Seizures were classified by using the modified Racine's scale [34] (1–5), animals were separated into “weak” and “strong” groups according to their seizure activity [28]. Seizure activity was defined for every animal by using the maximal value of Racine -scale reached more than once.

In the weak group mice developed only a few mild seizures characterized by few ictal seizure period in the EEG accompanied with startling and shaking, whereas in the strong group the seizures were frequent and EEG showed several ictal seizure periods behaviorally represented by intense motor symptoms including jaw movements, salivation, and forelimb clonus with rearing and tonic-clonic seizures [28], [34], [35].

Acute phase was examined 2 hours after pilocarpine injection. The period examined 1–3 days after the injection was regarded as the latent phase. At day three mass cell loss began, therefore this time point was considered as the end of the latent phase. EEG recordings were carried out 1 month after pilocarpine treatment in the chronic phase, at this time point recurrent seizures occurred in most of the strong epileptic animals.

We examined the expression pattern of CB1-Rs in the hippocampus at different survival times: 2 hours after the treatment in the acute phase, 1 and 3 days after the treatment in the latent phase and 1–2 months after the treatment in the chronic phase.

### *In vivo* electrophysiology

We proved the occurrence of recurrent electrographic seizures with EEG recordings in the chronic phase. Mice were anesthetized with a 1–1.5% halothane-air mixture (Narcotan, Leciva, Praha, Czech Republic) and secured in a stereotaxic frame (David Kopf, 900 USA, equipped with a SUPERTECH Ltd. made mouse adaptor HU).

Five holes were drilled into the skull above the frontal and parietal lobe bilaterally and above the cerebellum (reference). Stainless steel wire electrodes (MEDWIRE SST1) were placed on the skull and covered with conductive paste (Ten20, USA) to decrease the impedance. The electrodes and the connector were embedded in dental acrylic cement (GS, Japan). This way the electrodes were firmly implanted to the skull but the dura was not pierced. The EEG activity was recorded by a Grass EEG 8B model (Grass Instruments, Quincy, MA, USA), filtered at 1 Hz to 70 Hz and amplified (20 k). Data were recorded with a CED 1401 system using SPIKE2 v2.1 software (Cambridge Electronic Design Limited, Cambridge, UK). Sampling rate was 500 Hz, amplification 20 µV/mm, filter: LP: 70 Hz, HP: 1 Hz.

Recordings were made for 3 days, in the morning (9 AM) and in the afternoon (4PM) for 1.5 hours. The behaviour of the animal was observed during EEG recording. To answer the question whether animals had different diurnal and nocturnal epileptic activity, 24-hour continuous EEG monitoring was also carried out in 4 animals (2 controls, 2 chronically epileptic mice).

### Analysis of the EEG recordings

All EEG recordings were evaluated by three independent and experienced researchers to determine the occurrence of seizures or interictal spikes [36]. EEG recordings were carried out in 16 mice (6 controls, 4 weak and 6 strong epileptic) in the chronic phase. In 4 animals (2 controls, 2 chronically epileptic ones) 24-hours EEG monitoring was carried out.

Epileptic activity was considered as interictal burst when the amplitude of occasionally appearing EEG spike activity was three times higher than the normal resting control EEG waves. The ictal EEG was determined so that the appearance of large amplitude EEG spikes was continuous and that activity pattern lasted for at least 5 seconds. Occasionally, an increase of frequency and decrease of amplitude of large spikes were seen prior to the ictal event. That phase was considered as interictal activity. Data were analyzed with SPIKE2 v2.1 software (Cambridge Electronic Design Limited, Cambridge, UK).

### Slice preparation

Two hours after pilocarpine injection, control and “strong” epileptic mice (two of each) were deeply anesthetized with isoflurane (Abbott Labs, USA) After decapitation, the brain was quickly removed and placed into ice-cold artificial CSF containing (in mM): sucrose, 252; KCl, 2.5; NaHCO_3_, 26; CaCl_2_, 0.5; MgCl_2_, 5; NaH_2_PO_4_, 1.25; glucose, 10, and bubbled with 95% O_2_ and 5% CO_2_ (carbogen gas). We prepared 400- µm-thick coronal slices using a Leica (Nussloch, Germany) VT1000S microtome. Slices containing the hippocampal formation were trimmed from other brain regions. 2 slices of each animal were immediately transferred to fixative containing 0.05% glutaraldehyde (TAAB, UK), 4% paraformaldehyde (TAAB, UK) and 15% picric acid in 0.2 M phosphate buffer (PB), whereas other slices were kept in an interface-type holding chamber at room temperature for 2 hours, before fixation. After overnight fixation, CB1-immunostaining was carried out.

### Tissue preparation

Animals were sacrificed at different survival times including 2 hours (n = 22), 1 day, 3 days (n = 48), 1 month and 2 months (n = 105) after pilocarpine administration. Mice were perfused under equithesine anesthesia (chlornembutal 0.3 mL/100 g), first with physiological saline (1 min) and then with a fixative containing 0.05% glutaraldehyde (TAAB, UK), 4% paraformaldehyde (TAAB, UK) and 15% picric acid in 0.1 M phosphate buffer (PB) for 30 min.

### Immunocytochemistry

Brains were removed from the skull and 60 µm thick vibrotome sections were cut from the blocks. Following washing in PB (6×10 minutes), sections were cryoprotected in 30% sucrose for 1–2 days, followed by freezing three times over liquid nitrogen. Sections were processed for immunostaining against CB1-R (from Prof. M. Watanabe, Hokkaido University, Sapporo, Japan) (described by Fukudome et al., 2004 [37]), Neuronal Specific Nuclear Protein (NeuN) and Heat Shock protein 72 (HSP72) as follows. Sections were transferred to Tris-buffered saline (TBS, pH 7.4), then endogenous peroxidase was blocked by 1% H_2_O_2_ in TBS for 10 min. TBS was used for all the washes (3×10 min between each step) and dilution of the antisera. Non-specific immunostaining was blocked by 5% normal goat serum, 0.1 g/ml glycine, 0.1 g/ml lysine. A polyclonal guinea pig antibody against CB1-R (1∶1000), monoclonal mouse antibody against NeuN (1∶4000 Chemicon) and a monoclonal mouse antibody against HSP72 (1∶800, Calbiochem) were used for 2 days at 4°C. The specificity of the antibody has been thoroughly tested by the manufacturer and in our laboratory using CB1 knock out mice [38]. For visualization of immunopositive elements, biotinylated anti-guinea pig or anti-mouse immunoglobulin G (IgG) (1∶250, Vector, 2 hours) was applied as secondary antiserum followed by avidin-biotinylated horseradish peroxidase complex (ABC; 1∶250, Vector, 1.5 hours). The immunoperoxidase reaction was developed by 3,3′-diaminobenzidine tetrahydrochloride (DAB; Sigma) as a chromogen dissolved in TRIS buffer (TB, pH 7.6). Sections were then treated with 1% OsO_4_ in PB (40 min) and dehydrated in ethanol (1% uranyl acetate was added at the 70% ethanol stage for 40 min) and mounted in Durcupan (ACM, Fluka). The control sections were processed in the same way. For immunogold staining against CB1-R, the sections were blocked by 5% normal goat serum, 0.1 g/ml glycine and 0.1 g/ml lysine (GE Healthcare UK Limited). Incubation in anti-CB1 guinea pig antiserum (1∶1000, 2 days) was followed by a second blocking step with 5% normal goat serum, 0.1 g/ml glycine, 1 g/ml lysine and 0.1% fish gelatin. Secondary antiserum was ultra small gold conjugated goat anti-guinea pig (1∶50, overnight incubation, Aurion). Gold labeling was intensified using the R-Gent silver intensification kit (Aurion, Wageningen, The Netherlands). Sections were then osmicated (0.5% OsO_4_, 30 min, 4°C), dehydrated and embedded in Durcupan. The control hippocampi were processed in the same way.

After light microscopic examination, areas of interest were re-embedded and sectioned for electron microscopy. Ultrathin serial sections were collected on Formvar-coated single slot grids, stained with lead citrate, and examined with a Hitachi 7100 electron microscope.

### Gallyas silver impregnation

Degenerated neuronal elements were visualized with the staining procedure of Gallyas et al. [39]. The steps of this staining are the following: 2×5 min in the pretreating solution (2% NaOH and 2.5% NH_4_OH), 10 min in the impregnating solution (0–0.8% NaOH, 2.5% NH_4_OH, 0.5% AgNO_3_), 3×5 min in washing solution (0.5% Na_2_CO_3_ and 0.01% NH_4_NO_3_ in 30% ethanol), 1 min in developing solution (0.4–0.6% formaldehyde and 0.01% citric acid in 10% ethanol, pH 5.0–5.5), 3×10 min wash in 0.5% acetic acid. Afterwards sections were mounted on gelatin-coated slides, dehydrated in xylene and covered with XAM neutral medium (BDH, Poole, UK).

### Determination of principal cell loss

In the chronic phase, the severity of the acute seizures (using the Racine-scale, seizures from 1 to 5) was correlated with the cell-loss found in CA1, CA3 and hilus. Cell loss was determined in case of a large number of animals using a well defined semiquantitative scale by two independent examiners as shown previously [40], [41], [42]. Each region was classified as type 1–3 according to the severity of cell death (type1: cell-loss under 10%, type2: cell-loss between 10% and 50%, type3: cell-loss above 50%). Pearson correlation was used to calculate the relationship between cell-loss and seizure strength. Seizure strength was defined for every animal by using the highest number of Racine scale reached during acute seizures. This number, representative for each animal was correlated with the severity of cell death (types 1–3) in each subregion.

### Quantitative electron microscopic analysis

Electron microscopic analysis was carried out in the dentate gyrus, since this area is affected by seizure induced changes, however granule cells are well-preserved even in case of robust sclerosis in the CA1 [43], [44] therefore it is possible to examine the surviving elements which may not be the case in CA1. The quantity of symmetric and asymmetric synapses established by CB1-R-positive axon terminals was examined in the str. moleculare of 3 control, 3 chronic epileptic and 3 acute epileptic hippocampi. Serial sections were made from the blocks reembedded from stratum moleculare and examined in the electron microscope. CB1-R stained terminals were analysed in every 10th section in order, following the rules of systematic random sampling, to avoid sampling of the same axon terminals. Photographs were taken of all terminals in a given area, and the ratio of immunostained symmetric versus asymmetric synapses was determined. To quantify the exact changes in the number of stained terminals in the chronic and acute phase, we further examined the str. moleculare. The number of CB1-R immunostained terminals forming symmetric and asymmetric synapses was calculated, both in control and in epileptic samples and normalized. The studied areas were measured with NIH ImageJ (U.S. National Institutes of Health, Bethesda, MD) program and were normalized to unit area (40 000 µm^2^).

To assess any changes occurring in the density of CB1-Rs per terminals, the number of gold particles located in the membrane of axon terminals was counted and normalized to a unit length of the terminal's perimeter (perimeter length was measured with NIH ImageJ, number of particles was normalized to 1 µm). Significance was tested using Mann-Whitney U-test (Statistica 6.0).

## Results

### The pattern of cell loss in pilocarpine induced epilepsy

**Figure 1:**
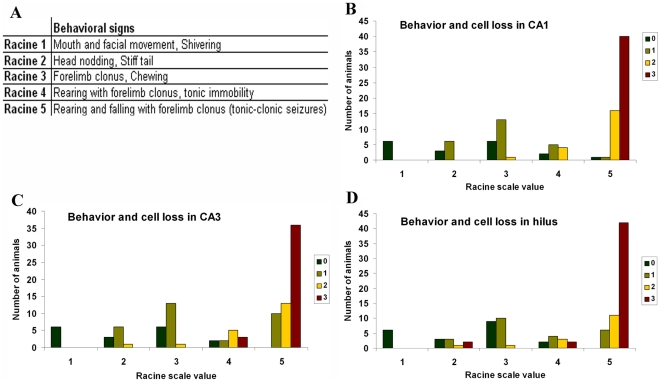
Table A describes seizure behavioral scoring for each Racine scale value. Graphs B, C and D show the correlation between seizure intensity (Racine scale value) and cell loss (color code indicates cell loss from 0 to 3, where 0 refers to no cell loss, 1: cell-loss under 10%, 2: cell-loss between 10% and 50%, 3: cell-loss above 50%) in CA1, CA3 and the hilus respectively. Correlation between Racine scale value and cell loss in the regions proved to be significant using Pearson correlation (p<0.05).

**Figure 2:**
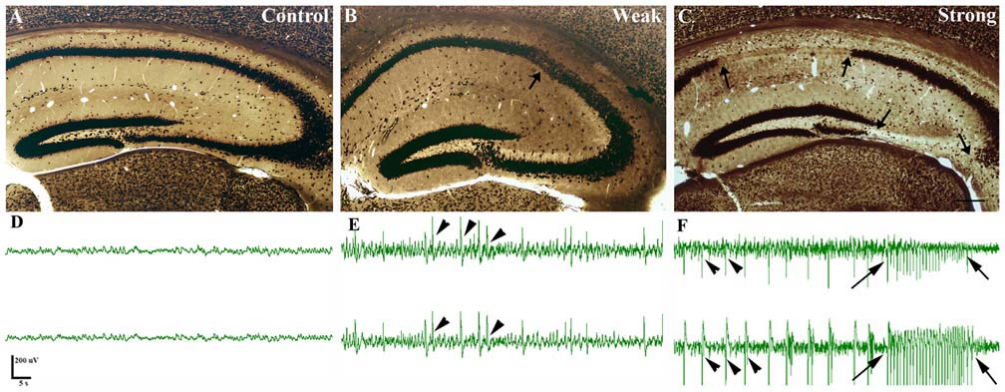
Anatomical and physiological changes in pilocarpine induced epilepsy. Light micrographs of control (A) and epileptic (B, C) animals immunostained for NeuN. Animals survived for 1 month after pilocarpine treatment. Compared to control mice (A), in non-sclerotic “weak” animals (B) only mild, restricted changes (arrow) are seen occasionally in the hippocampus. In the sclerotic hippocampi of “strong” animals (C) a characteristic pattern of neuronal damage appears. The principal cell loss is over 50% (sclerosis) mostly in CA1 and CA3 (arrows) and in the hilus. In the EEG recordings of control animals no ictal or interictal acitivity can be seen (D), however, in weak animals (E) interictal spikes (arrowheads) occur frequently. In strong animals (F) recurrent seizures are observed in most cases, both interictal and ictal (arrows) activity appeares. An increase in frequency and decline in amplitude can be seen immediately preceding and after the seizures. Scale (A, B, C): 200 µm.

As a first step, we carried out detailed investigations to clarify the alterations of the hippocampal circuits on the pilocarpine model of epilepsy using CD1 mice. Based on the behavioral signs during the acute seizures, animals were classified as “weak” or “strong” epileptic using the modified Racine scale [28], [34], [35]. Animals showing seizure intensity from Racine 1 to Racine 4 (shaking, chewing, nodding, forelimb clonus, rearing but no tonic-clonic seizures) were assigned to the weak group. However, in the strong group all animals showed strong tonic-clonic seizures (Racine 5) with other less severe seizure manifestations (**Fig. 1**). We examined the cell loss pattern in the hippocampi at different survival times using NeuN-immunostaining (**Fig. 2**)

In the acute phase (2 hours after the treatment) cell loss was observed neither in the weak nor in the strong animals (data not shown).

In the latent phase (1–3 days post-pilocarpine) loss of sensitive interneurons (calretinin positive) and few principal cells could already be seen in the CA1 and CA3 regions (3 days) (not shown). Three days post pilocarpine, cell loss was usually patchy in the CA1-3; occasionally pyramidal cells disappeared in larger areas.

In the chronic phase patchy cell loss was found infrequently in “weak” animals, but none of their hippocampi showed sclerosis (**Fig. 2B**). In contrast, hippocampi of the “strong” mice showed sclerosis in most cases (70%) meaning that CA1 and CA3 regions were shrunken, atrophic and more than the half of the cells were missing from the CA1 region. Loss of vulnerable interneurons was also observed in all regions (**Fig. 1**) (**Fig. 2C**).

Based on the severity of cell death, the degree of damage of subregions (CA1, CA3, dentate gyrus) was classified as type 1 (mild cell loss), type 2 (patchy cell loss) and type 3 (sclerotic), defined according to the following semiquantitative scale: type 1, up to 10% of the cells are damaged; type 2, 11–50% of the cells are missing; type 3: more than 50% of the cells are missing. CA1 and CA3 were considered sclerotic when cell loss exceeded 50%, especially when strata oriens, pyramidale and radiatum could not be separated any more [40], [41].

Statistical analysis revealed a significant correlation (Pearson correlation) between the degree of cell-loss and seizure strength in Racine scale values (p<0.05) (105 animals) (**Fig. 1**). Thus, behavioral signs of the acute seizures could be used to predict the degree of cell loss in the chronic phase.

### *In vivo* electrophysiological recordings

To prove the appearance of epileptic activity and recurrent seizures, EEG recordings were carried out in 16 mice (6 controls, 4 weakly and 6 strongly epileptic) in the chronic phase. On the basis of these recordings, the EEG activity of pilocarpine treated mice differed from controls (**Fig. 2D**). Strong synchronization and numerous interictal spikes were seen separately or in clusters. In members of the weak group (**Fig. 2E**) recurrent seizures were rarely seen (in 1 mouse out of 4), while interictal spikes (incidence 2.2±2.26 Hz, amplitude 581±160 µV) occurred in all cases. However, in members of the strong group recurrent seizures appeared (ictal spikes with a frequency of 5.9±2.3 Hz, amplitude 546±112 µV) in nearly every animal (5 out of 6) (**Fig. 2F**). Thus, the occurrence of recurrent seizures in the chronic phase showed strong association with the severity of acute seizures.

One month after pilocarpine injection in 4 animals (2 controls, 2 strong) 24 hours long EEG monitoring was carried out to investigate nocturnal epileptic activity. We observed seizure activity in the EEG represented by spikes in all animals. No animals showed exclusively nocturnal seizures, but they performed recurrent seizures during the whole day. Seizure incidence was the highest in the afternoon compared to other periods of the day.

### HSP72 expression in the latent phase of epilepsy (1, 3 days post pilo)

To examine whether anatomical differences occur at an early time point between animals with strong or weak seizures, immunostaining against Heat Shock Protein 72 (HSP72) was carried out to visualize cells suffering from excitotoxic damage, since this protein confirms the emergence of abnormal proteins due to various damaging effects [41], [45], [46].

**Figure 3:**
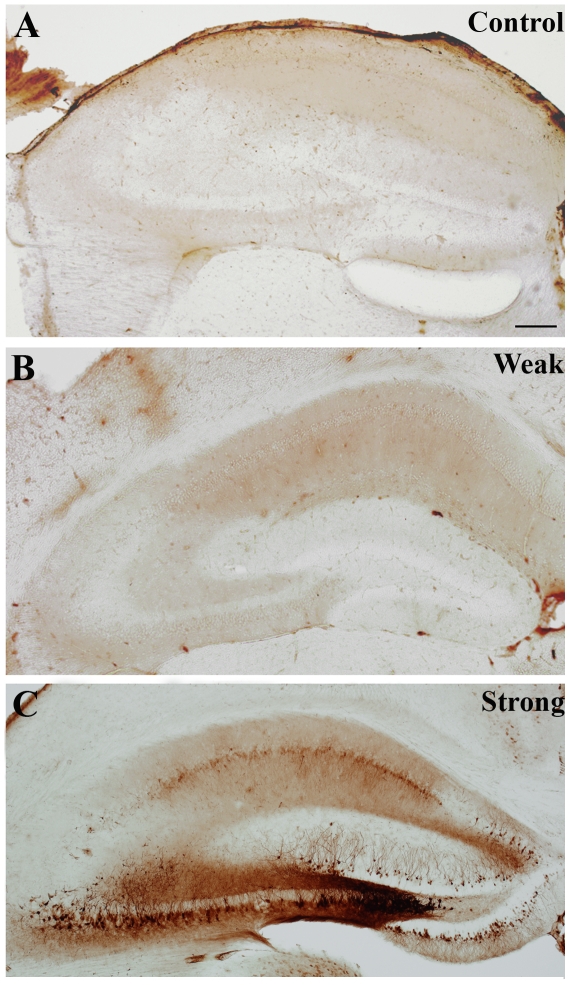
Demonstration of excitotoxic insult 1 day after pilocarpine treatment. In animals of the “weak” group (B) HSP72-immunostaining is control-like (A), only a slight increase in background-staining can be seen, but no stained cells with processes appear. Although there are no morphological signs of neuronal degeneration at this early stage in the hippocampi of “strong” epileptic animals (C) either, the vulnerable cells as well as the resistant granule cells start to express HSP72, and are stained in a Golgi-like manner. Faint homogeneous staining of CA1 pyramidal cells can also be seen. Scale: 200 µm.

In control hippocampi (**Fig. 3A**) immunostained cells were never seen, light background staining appeared occasionally in the layers of principal cells.

In the acute phase of epilepsy (2 hours after injection) no significant alteration was observed with HSP72 immunostaining, the hippocampi of both weak and strong animals were control-like (data not shown).

In the latent phase positive cells were not seen in weak epileptic animals (**Fig. 3B**), however, in members of the strong group robust changes occurred 1 day after the induction of the seizures (**Fig. 3C**). Principal cells with Golgi-like HSP-staining appeared in the str. pyramidale of the CA3, and among the granule cells in the dentate gyrus. Occasionally immunopositive cells were seen in the CA1 str. pyramidale as well.

**Figure 4:**
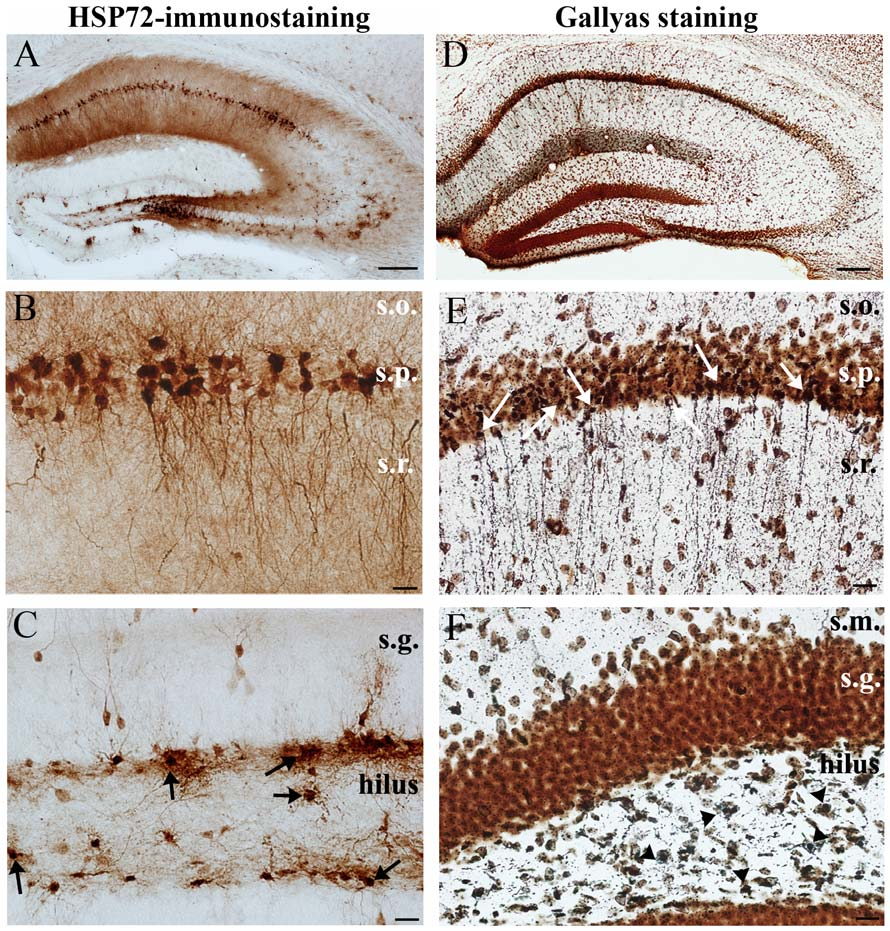
Cell loss and degeneration at day 3 post pilocarpine. HSP72 is heavily expressed in a large population of pyramidal cells (A, B) and in some mossy cells, indicating that they were exposed to excitotoxic insult. In contrast, hardly any granule cells are stained (arrows) (C) compared to 1 day post SE. Gallyas silver impregnation reveals that in the early latent period principal cell loss begins at a large scale (orange cells are alive). Dark silver deposit is present in neurons irreversibly damaged (D, E, F). Argyrophilic cell degeneration can be seen in the vulnerable regions of the hippocampus, like the CA1 pyramidal cell layer (E) and the hilar region (F) (arrows in the CA1, arrowheads in the hilus). (s.o.: str. oriens, s.p. str. pyramidale, s.r.: str. radiatum, s.m.: str. moleculare, s.g.: str. granulosum) Scale: A, D 200 µm, B, E, F, C and F: 50 µm.

Three days after pilocarpine treatment weak samples displayed the same appearance seen in controls (not shown), with a modest increase in background staining. In contrast, in the hippocampi of strongly epileptic animals a marked increase in HSP72 expression was found in CA1 (**Fig. 4 A, B**) and CA3 (mostly CA3c) pyramidal cells, and in the mossy cells of the hilus (**Fig. 4 C**). However, three days post pilocarpine fewer granule cells were stained compared to samples 1 day after pilocarpine.

These results prove that cellular differences can be seen between animals with different acute behaviour; validating our classification into “weak” and “strong” groups. Irreversibly damaged cells were visualized with Gallyas silver impregnation (**Fig. 4D–F**). One day after pilocarpine injection dark silver deposit was present in hilar neurons undergoing agyrophilic degeneration (not shown). Three days after pilocarpine silver accumulation appeared in additional subregions; besides strata pyramidale and radiatum of the CA1 (**Fig. 4E**) degenerating somata and dendrites appeared in the hilus (**Fig. 4F**).

### Analysis of CB1-R distribution in different phases of pilocarpine-induced epilepsy

#### Distribution of CB1 receptors in control tissue

**Figure 5:**
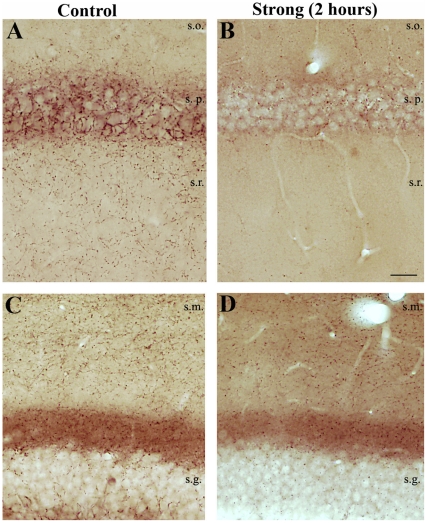
Changes of CB1-R-staining in the acute phase. In control samples intense staining of CB1-R-positive fibers can be seen in CA1 stratum pyramidale and radiatum (A) and in the molecular layer of the dentate gyrus (C). In the acute phase of epilepsy (2 hours after pilocarpine injection) the hippocampi of strongly epileptic animals show decrease of CB1-R staining (B, D) compared to controls (A, C). The change is more remarkable in CA1 than in the DG, where the faint diffuse staining of the inner str. moleculare is hardly affected. Scale: 50 µm.

In control samples intense CB1-R immunostaining was found throughout the hippocampus. Immunopositive cell bodies of interneurons were seen in all hippocampal subfields, mostly in strata radiatum and lacunosum-moleculare of CA1 and CA3, as well as at the border of the hilus and in the inner molecular layer of the dentate gyrus. Intense staining of CB1-R-positive fibers was found in stratum pyramidale of the cornu Ammonis (**Fig. 5A**) and in the molecular layer of the dentate gyrus (DG) (**Fig. 5C**). In contrast, less dense labeling was observed in the strata radiatum (**Fig. 5A**) and granulosum (**Fig. 5C**) [38], [47], [48].

At the electron microscopic level numerous immunopositive axon terminals were found forming symmetric or asymmetric synapses. Immunogold particles indicating the presence of CB1-Rs was located predominantly perisynaptically as described earlier ([49], [50]. Glial cells and processes were not immunopositive either in control or in epileptic tissue.

#### Distribution of CB1 receptors in the acute phase of epilepsy (2 hours)

In the acute phase, epileptic hippocampi from members of the weak group showed control-like phenotype: no major changes were seen in the distribution and density of immunolabelled elements. In contrast, in the strong group a robust decrease in immunopositivity was observed throughout the hippocampus, namely, the dense axonal meshwork seen in the strata moleculare, radiatum and oriens of controls was substantially reduced (**Fig. 5B, D**). Moreover, CB1-R immunopositive boutons forming baskets in the principal cell layers could hardly be seen at light microscopic level (**Fig. 5B**).

#### Reversible changes in CB1-R expression in acute slices (2 hours post pilo)

**Figure 6:**
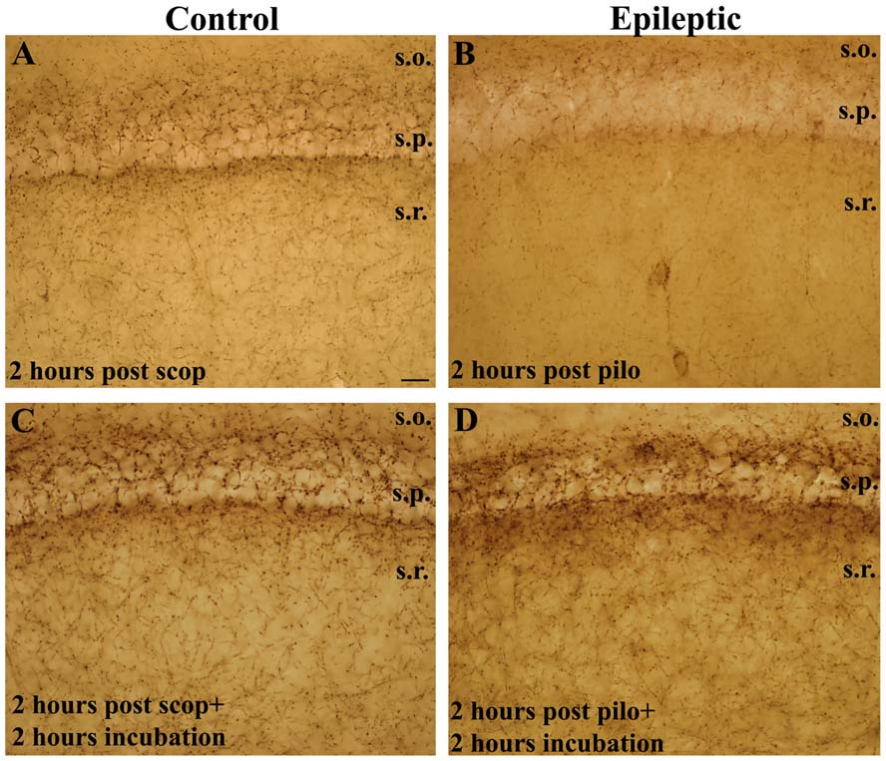
Reversible changes in CB1-R-staining in slice preparation (acute phase). In CA1 region of surviving brain slices 2 hours post pilocarpine a massive decrease is seen in CB1-R staining in strongly epileptic animals (B) compared to scopolamine treated controls (A) as it was seen in perfusion-fixed epileptic animals. In contrast, the slices from the same animals which were incubated in ACSF for an additional 2 hours the CB1-R staining in the epileptic tissue (D) did not differ from control slices (C). Scale: 50 µm.

To investigate the electrophysiological correlates of our anatomical findings observed in acute phase, we planned to record the effects of CB1–R activation on hippocampal function. *In vitro* slices were prepared from the hippocampi of control and strongly epileptic animals. As a first step, immunostaining for CB1-Rs was carried out on the slices at two time points: immediately fixed after the slice cut and after 2 hours of incubation in an interface-type holding chamber. Surprisingly, we observed that after 2 hours of incubation, slices from strongly epileptic animals showed control-like distribution of CB1-Rs (**Fig. 6A, C, and D**). However, when slices from the same animals were immediately fixed after cutting, a similar decrease in CB1-R distribution was found as seen in perfusion fixed animals compared to controls (**Fig. 6B**).

These data suggest that under our circumstances the consequences of the acute phase on hippocampal CB1-R function cannot be studied using in vitro slice preparations, since the changes in CB1-R distributions after acute seizures recovers within the incubation time that has to precede recording.

Since functional consequences of CB1-R decrease could not be established with *in vitro* methods, we studied the seizures of animals *a priori*, lacking CB1-Rs.

#### Increased mortality after seizures in CB1 KO animals

**Figure 7:**
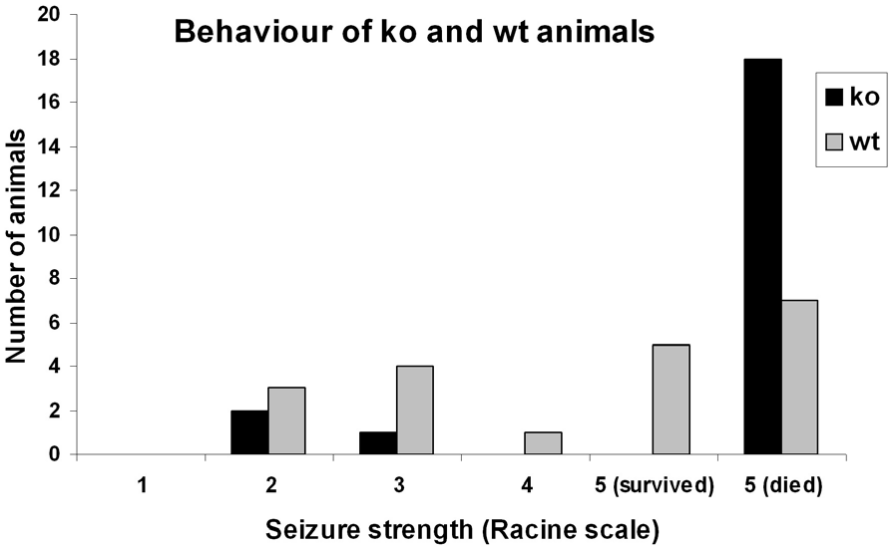
Different behaviour and survival of knock out and wild type animals. Graph showing the number of animals as a function of seizure strength demonstrates that knock out animals had more severe seizures compared to wild types and never survived tonic-clonic (Racine 5) seizures.

We addressed the question how seizure susceptibility changes in CB1-R knock out mice, therefore 22 controls were examined, 22 CB1-R knock out animals and 21 wild type littermates were injected with pilocarpine in a different set of experiments. CB1-R KO animals received very intense seizures (typical for strong epileptic animals) they were more susceptible to epilepsy as only 3 animals had mild seizures out of 22 and all 19 animals showing strong seizures died in 15 minutes (**Fig. 7**). Mice receiving only mild or no seizures (members of the weak group), survived and were sacrificed in the chronic phase. These animals showed no signs of anatomical alterations, NeuN and Gallyas labeling was similar to the staining in weak wild type littermates (not shown). These results may suggest a protective role of CB1-R activation in epilepsy as it has been shown previously [20], [51], [52].

#### Ultrastructural changes of CB1-R expression in the acute phase of epilepsy

At the electron microscopic level occasionally degenerating profiles were observed throughout the hippocampus. They were mostly oedemic dendritic profiles, axon terminals and spines; however, they were not selectively CB1-R-positive. To quantify the changes, systematic random sampling was carried out. We could not take advantage of dissector method, since one cannot obtain accurate counts in a reasonable time when structures of interest form a very small fraction [53]. Moreover, we wished to minimize alterations caused by the sprouting of non-positive fibers in the examined area, therefore, we have examined a large area (>40.000 µm2) in a single plane.

**Figure 8:**
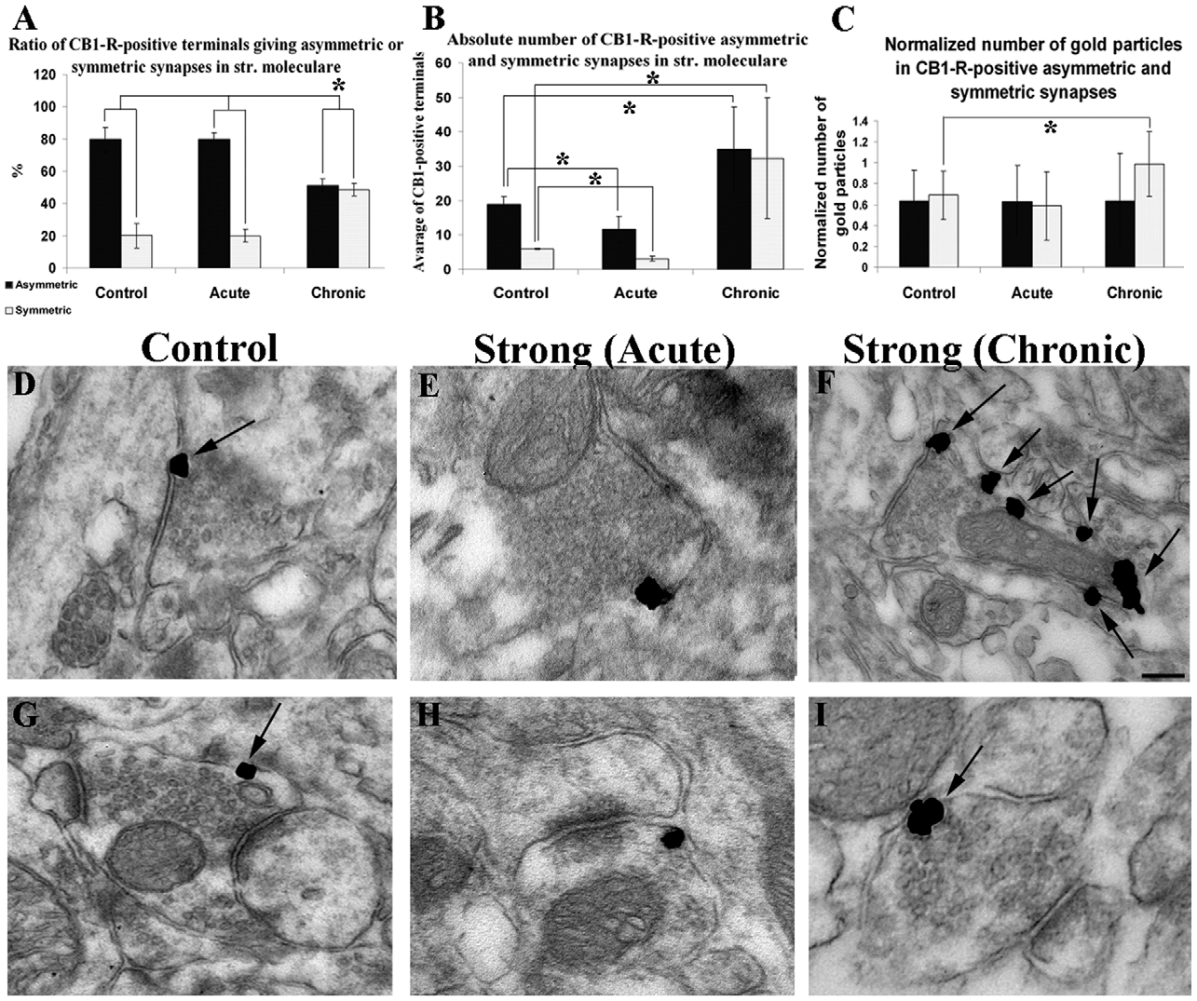
Quantitative changes of CB1-Rs in the acute and chronic phase of epilepsy. Graph **A** shows changes in the ratio of CB1-R immunopositive terminals establishing symmetric versus asymmetric synapses in control and epileptic tissue in str. moleculare of the DG. No difference was found in the hippocampi of strong animals in the acute phase compared to controls. However, in strong animals in the chronic phase a significant increase occurred in the ratio of stained symmetric versus asymmetric synapses. Graph **B** shows changes in the absolute number of symmetric versus asymmetric synapses in control and epileptic tissue. Significant decrease was found in the number of stained asymmetric and symmetric synapses in the hippocampi of strong animals in the acute phase, compared to controls. In contrast, in strong animals in the chronic phase, the number of immunostained asymmetric and symmetric synapses significantly increased. Graph **C** shows changes in the normalized number of gold particles in control and epileptic tissue. No difference appears in the mean quantity of gold particles in asymmetric synapses in control or in epileptic tissue (acute or chronic phase). No difference was seen in case of symmetric synapses between the hippocampi of control and acute epileptic mice either. However, in chronically epileptic tissue the number of gold particles located in symmetric terminals increased significantly. Statistics were calculated with Mann-Whitney test (p<0.05). High power electron micrographs of CB1-R immunolabelled axon terminals from the str. moleculare of the DG of controls (D,G) acute and chronic animals 2 hours (E, H) and 1month (F, I) post pilo. Our antibody labels both CB1-R-positive terminals giving symmetric (D, E, F) and asymmetric (G, H, I) synapses. Gold particles are located extrasynaptically. In the chronic, strong tissue the number of gold particles on terminals forming symmetric synapses has increased (F). Scale: 200 nm.

To confirm changes caused by swelling we measured the perimeter of immunopositive terminals. A total of 298 terminals were digitized (169 of control tissue, 129 of strong epileptic tissue) and analyzed. Compared to controls a significant increase in perimeter (p<0.05; Mann-Whitney test) was found in case of terminals forming symmetric synapses (control: 2±0.67 µm, strong epileptic: 2.63±0.87 µm) however, no such difference was observed among stained terminals forming asymmetric synapses (control: 1.9±0.76 µm, strong epileptic: 2.12±0.9 µm). Afterwards we compared the ratio of immunolabelled axon terminals establishing symmetric versus asymmetric synapses in the same sample of 298 terminals (**Fig. 8A**). The percentages of CB1–R immunopositive axon endings forming symmetric and asymmetric synapses found in acute epileptic samples (**Fig. 8E,H**) were calculated and compared to the percentage observed in controls (**Fig. 8D,G**). The analysis revealed no significant change in these ratios (control: 79.6±7.6% in case of asymmetric and 20.4±7.6% in case of symmetric synapses; strong epileptic: 80±4% in case of asymmetric and 20±4% in case of symmetric synapses, p>0.05; Mann-Whitney test) (**Fig. 8A**). To uncover changes in the overall number of stained terminals, we counted every stained terminal in a given area of str. moleculare, and normalized the results to unit area (40 000 µm^2^). Compared to control tissue, a significant decrease was found in the number of labeled asymmetric and symmetric synapses (control asymmetric: 18.9±2.3; symmetric: 6±0.2; strong epileptic asymmetric: 11.8±3.7, symmetric: 3.2±0.7; p<0.05; Mann-Whitney test) (**Fig. 8B**). Taken together, the results imply that mechanism(s) other than axon terminal degeneration could account for the loss of CB1-R staining in the acute phase of epilepsy.

To understand further changes in CB1-R expression we examined other phases as well.

#### Distribution of CB1 receptors in the latent phase of epilepsy (1 and 3 days post pilo)

One day after pilocarpine injection CB1-R levels were control-like both in weak and strong animals. Three days after pilocarpine injection upregulation of CB1-R immunoreactivity occurred in some animals of the strong group. There was a gradual recovery in CB1-R intensity 1 and 3 days post pilo. 3 days after SE principal cells began to degenerate (**Fig. 4**) and HSP72 staining was more extended referring to excitotoxic damage caused by the initial seizures. However, in the latent phase the decreased intensity of the receptor staining was not observed any longer. CB1-R staining proved to be control-like or moderately increased.

#### Distribution of CB1 receptors in the chronic phase of epilepsy (1 and 2 months)

**Figure 9:**
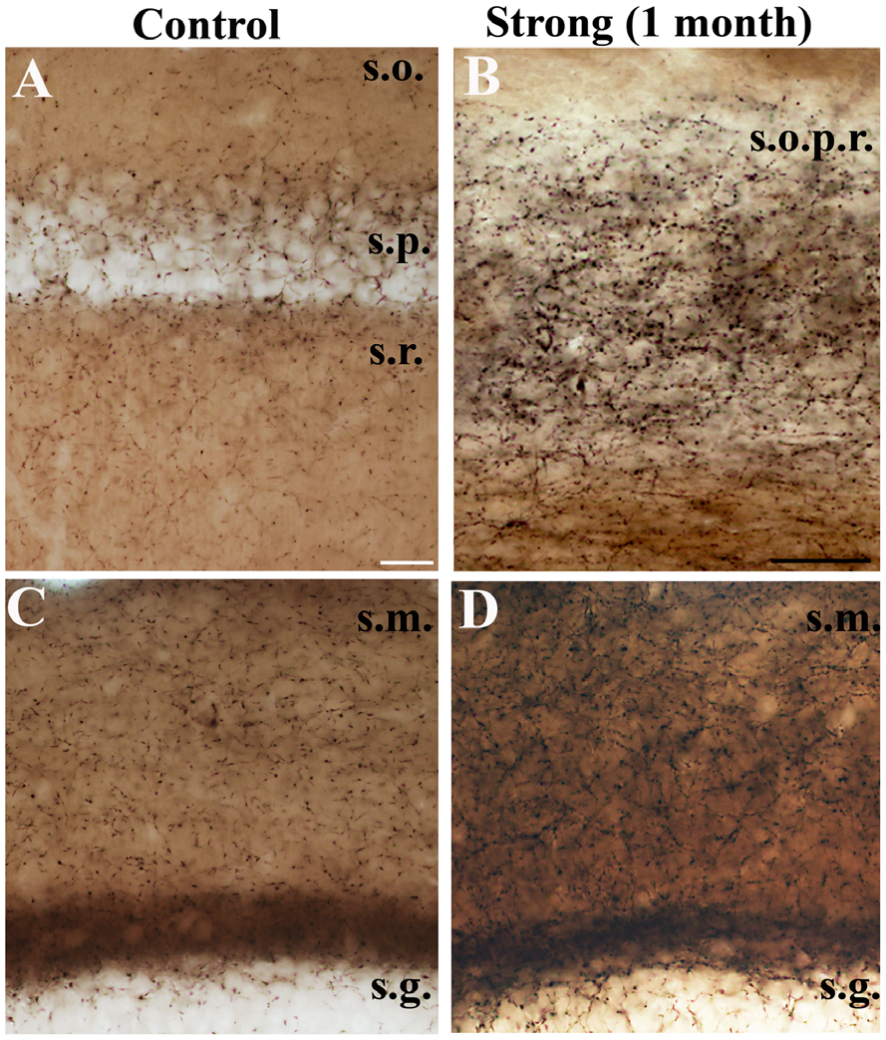
Changes in CB1-R-staining in the chronic phase. CB1-R immunostaining in the hippocampi of control (A, C) and chronically epileptic, sclerotic mice (1 month post pilocarpine)(B, D). In the sclerotic samples the general CB1-R immunostaining is much stronger throughout the hippocampus. In the CA1 (A) a dense immunostained axonal plexus can be seen in the sclerotic CA1 region (layers cannot be distinguished) (B). In the dentate gyrus stained fibers establish more dense meshworks (D). Scales: A, C, D: 50 µm B: 20 µm.

To study the long-term changes in CB1-R levels, their distribution was examined one month after pilocarpine injection, in the chronic phase. In general, similar alterations in CB1-R staining were found in these animals as we observed earlier with antibody recognizing only CB1-R at inhibitory terminals (Magloczky et al., 2010) (**Fig. 9A, C**). In epileptic animals of the weak group the distribution of CB1-Rs was mostly control-like. Occasionally, a highly restricted upregulation appeared in str. pyramidale of CA1. In the sclerotic samples the general CB1-R immunostaining was enhanced throughout the hippocampus. The density of immunostained fibers in CA1 increased heavily in surviving elements of strata pyramidale and radiatum (**Fig. 9B**). Similarly, in DG a dense CB1-R-positive axonal plexus was found in strata moleculare and granulosum (**Fig. 9D**). Immunopositive interneuron somata were present both in the dentate gyrus and in the CA1 and CA3 areas.

#### Ultrastructural analysis of CB1-R distribution in the chronic phase

At the electron microscopic level, numerous glial elements and occasionally degenerating profiles were seen, changes that are typical for epileptic tissues [2], however glial elements were not positive for CB1-R staining. The general ultrastructural features of CB1-R-positive elements were unchanged; nevertheless, the number of stained terminals increased significantly (p<0.05) (**Fig. 8A, B**). Changes in the ratio of stained terminals establishing symmetric versus asymmetric synapses were analyzed (169 controls, 178 strong epileptic). In epileptic animals the ratio was significantly changed; the mean percentage in control tissue was 79.6±7.64% in case of asymmetric (**Fig. 8G**) and 20.4±7.6% in case of symmetric synapses (**Fig. 8D**), in strong epileptic tissue 51.4±3.9% of the examined terminals proved to be asymmetric (**Fig. 8I**) whereas 48.6±3.9% was symmetric (**Fig. 8 F**) (p<0.05; Mann-Whitney test) (**Fig. 8A**). These results may suggest either a loss of stained asymmetric synapses or an increase of symmetric synapses, or both.

To address this question, we used systematic random sampling as described above and digitized every stained terminal in a given area of str. moleculare, and normalized the results to unit area (40.000 µm^2^). Compared to control tissue a significant increase was found in the number of CB1-R-positive asymmetric and symmetric synapses (control asymmetric: 18.9±2.3 symmetric: 6±0.2; epileptic: asymmetric: 34.9±10.9, symmetric: 32.3±18.5, p<0.05; Mann-Whitney test), (**Fig. 8B**). During the analysis of immunogold-labeled axon terminals, in certain terminals we noticed an increase in the number of immunogold particles in the hippocampi of chronically epileptic animals of the strong group (**Fig. 8**).

To quantify these changes, immunogold particles were counted in 302 (125 controls and 177 strong epileptic) terminals. The number of gold particles in the membrane of CB1-R stained axon terminals forming symmetric or asymmetric synapses was counted and normalized to unit perimeter of the axonterminal membrane (particle/1 µm). No difference was found between asymmetric synapses in control and epileptic tissue in the average quantity of gold particles (control: 0.64±0.27, strong epileptic: 0.633±0.46, p>0.05, Mann-Whitney test). In contrast, the number of immunogold particles significantly increased in axon terminals forming symmetric synapses (control: 0.69±0.29, strong epileptic: 0.99±0.49, p<0.05, Mann-Whitney test) (**Fig. 8C,F**). No similar change was observed in tissue samples derived from strong animals in the acute phase neither in asymmetric synapses (control: 0.64±0.27, strong epileptic: 0.63±0.33) nor in case of symmetric synapses (control: 0.69±0.29, strong epileptic: 0.59±0.34 (**Fig. 8C**). In addition, the perimeter of immunopositive terminals establishing symmetric synapses significantly increased in strong epileptic animals in the chronic phase (control: 1.99±0.67 µm, strong epileptic: 2.7±0.9 µm). No such change was observed in terminals establishing asymmetric synapses (control: 1.89±0.8 µm, strong epileptic: 2.22±0.93 µm).

In summary, we found that the number of immunolabelled axon terminals forming asymmetric and symmetric synapses increased in the chronic phase. In addition, the number of gold particles indicating the presence of CB1-Rs was increased on terminals forming symmetric synapses. These results may indicate an elevation of CB1-R function in the chronic phase, which may act as an extremely powerful circuit-breaker on GABAergic and glutamatergic transmission (Katona&Freund, 2008).

## Discussion

In our model various aspects of epileptic cell loss and reorganization are similar to that seen in human TLE [42], [54], [55], [56], [57], [58]. On the basis of our results the strength of seizures in the acute phase can be used to predict future changes (e.g. cell loss) in the chronic phase. The vulnerability of cells differed between “weak” and “strong” groups, as indicated by HSP72-expression in the latent phase. The expression of CB1-Rs related to the GABAergic and glutamatergic axon terminals was strongly decreased without specific degeneration in the acute phase, but in the chronic phase a significant upregulation occurred in the number of immunostained terminals. In addition, a significant increase was seen in the number of CB1-Rs in a single terminal, but only in terminals forming symmetric synapses.

Endocannabinoids as retrograde signal molecules are released by large intracellular Ca^2+^ transients, complex-spike burst-firing, and/or phospholipase C activation via metabotropic receptors in neurons [8], [59]. They bind to presynaptic CB1 receptors located on glutamatergic and GABAergic axon terminals, and thereby decrease transmitter release from excitatory and inhibitory boutons arriving primarily onto the same neurons [13], [14].

The question arises whether the different changes in the endocannabinoid-mediated reduction of GABA and glutamate release [60], [61] contribute to seizure generation and maintenance, or should it rather be considered as a neuroprotective reaction [20], [52].

### Changes in the acute phase of epilepsy

Earlier investigations of short term changes in the endocannabinoid system revealed a robust downregulation in the acute phase [29], [30]. In these studies the loss of staining was explained by initial cell death or the degeneration of terminals. In contrast, in our samples mild degeneration was observed involving different profiles which were rarely CB1-R-positive; moreover, swelling was observed in case of immunostained terminals forming symmetric synapses. In addition, in our study the acute phase was examined 2 hours after pilocarpine injection. Previous studies examined the “acute” phase at e.g. 9 hours to 1 week post SE [30]. We were able to examine the acute changes as early as 2 hours post SE since we did not use benzodiazepines to terminate seizures. This way we could demonstrate that acute changes of CB1-R distribution are independent of cell loss and likely the consequence of initial seizure activity.

Furthermore, the decrease of CB1-Rs in acute slices proved to be a reversible change, since they were redistributed in the membranes in a short time after “ending the seizures”/sacrificing the animals. Therefore, *in vitro* electrophysiology may not suitable to examine functional changes in the acute phase. These examinations suggest the occurrence of internalization and recycling [62], or degradation shortly followed by de novo synthesis.

Endocannabinoids are known to be synthesized in an activity-dependent manner [8], [63], [64]. In case of epileptic hyperexcitation an increase may occur in endocannabinoid levels as proposed previously and shown in kainic acid model of TLE [19], [23], [31], [65], [66], [67], thus CB1 receptors become strongly activated. Although CB1 receptor agonists are potent anticonvulsants both in animal models and human patients with TLE [18], [20], [68], their constant presence may cause internalization of the receptor [69], [70] or changes in conformation (forming homo-or heterodimers) [71], [72] leading to proconvulsive effects [21], [22]. Therefore, the controversial effects of cannabinoid agonists may be explained by the timing of their application [25].

### Changes in the chronic phase of epilepsy

In the chronic phase of epilepsy a massive increase of CB1-staining located both at symmetric and asymmetric synapses was found throughout the hippocampus. Our results differ from that of Falenski et al [73] and Wyeth et al. [30], since in these studies the loss of staining was sustained throughout the chronic phase as well, at least in certain hippocampal subregions. This discrepancy could be explained by differences between the models. In our study we examined sclerotic animals with a cell loss pattern similar to that seen in human TLE patients. In animal models described earlier [30], [73] different results were found depending on the model conditions (e.g. termination of seizures by benzodiazepines or severity of cell loss pattern).

#### Sprouting of excitatory fibers

Increased number of glutamatergic terminals with CB1-Rs was found in the hippocampi of sclerotic (strong epileptic) animals, most probably due to the sprouting of mossy cell axons or CB1-R expressing subcortical fibers. This highlights the importance of endocannabinoid mechanisms in reducing glutamate release during epilepsy [19], [20]. Recent findings show that the overexpression of CB1-Rs on glutamatergic synapses can protect against excitotoxic damage [52]. Moreover, increased effects of CB1-R agonists and elevated levels of receptor protein were shown in the dentate gyrus of pilocarpine treated mice [51].

Controversially, the innermost part of stratum moleculare was examined previously by Ludanyi et al. showing that a downregulation of CB1-Rs related to glutamatergic terminals occurs in the inner molecular layer of the dentate gyrus in human TLE patients [27]. In the study of Ludanyi et al. the ratio of CB1-R stained asymmetric synapses was calculated by comparing the number of stained terminals to the number of unstained terminals in a given area. However, intensive sprouting of excitatory axon terminals in the stratum moleculare occurs in the epileptic tissue as it was described earlier (Magloczky et al. 2000; [1], [74], [75]. The increased amount of CB1-R unstained terminals may explain the decreased ratio of CB1-R-positive boutons found by Ludanyi et. al. Another reason for this discrepancy can be the different method for quantification. In our study exclusively CB1-R-postive terminals were quantified in a large area of str. moleculare.

In addition, a recent study showed that an increased CB1-R availability could be observed in human TLE, which correlated negatively with the latency following the last seizure [26], therefore CB1-R expression seems to be regulated very dynamically, which may easily explain differences between the results.

#### Sprouting of inhibitory fibers, changes in perisomatic inhibition

In the chronic phase of epilepsy CB1-R-positive (and also CCK-positive) cell bodies were preserved in the dentate gyrus and in the CA1 area, despite the mass principal cell loss. Enhancement of the immunostained terminal density deriving from the surviving CB1-R-positive interneurons was associated with the degree of cell loss [27], [28].

In case of CB1-Rs located in terminals establishing symmetric synapses a strong increase in CB1-R-immunostaining was found both in the hippocampi of epileptic patients and in mice with CA1 sclerosis [28]. Moreover, we found an increase of CB1-R level on single GABAergic terminals in parallel with an increase of terminal perimeter. Results of Chen et al. [76] show a chronic increase of CB1-Rs on axons of cholecystokinin-containing inhibitory cells following febrile seizure-like events. Our previous study, showing the sprouting of CB1-R-expressing interneuronal fibers and the elevation of CB1-R levels both in a chronic model (pilocarpine) and in human patients highlights the involvement of the reorganized endocannabinoid system in the chronic phase of temporal lobe epilepsy [28]. In addition, the increase in the size of inhibitory terminals may have a role in sustaining a more effective inhibition as proposed previously [77], [78].

Sprouting of CB1 expressing neurons related to GABAergic fibers (mostly CCK-containing cells [17] may have controversial effects depending on the depolarizing or hyperpolarizing effect of GABA. If GABA is hyperpolarizing, proconvulsant effects of CB1-Rs could occur, by reducing GABAergic inhibition [24]. However, in case of altered ion homeostasis, GABA_A_ receptors may have depolarizing effects [81], [82], [83], [84], [85], [86], [87], thus reduced GABA release would be anticonvulsive.

GABA release causing hyperpolarization may be anticonvulsant as well, since enhanced perisomatic inhibition in chronic epilepsy was proposed to have a role in synchronizing seizure activity [42], [58], [76], [79], [80]. In addition, in epileptic tissue the axons of perisomatic targeting interneurons often sprout, suggesting an enhancement in this type of inhibition [42], [80].

Consequently, the effect of altered CB1-R distribution may depend on the current network state and ion homeostasis. Reduced transmitter release may decrease seizure intensity in case of glutamate release, and also in case of GABA release, when GABA has a hypersynchronizing or depolarizing effect. These scenarios, if they occur during epileptic seizures, may lead to an antiepileptic network effect of the increased density of CB1-Rs.

### Conclusions

We showed that early seizures can determine the severity of future epileptic events. With the neurochemical marker HSP72 different mechanisms were shown to activate in strong and weak animals leading to profound differences also in the chronic phase.

In case of CB1-R-staining, robust changes were only found in animals with strong acute seizures. In animals with milder seizures and hardly any cell loss, changes in CB1 distribution remained undetectable with the current methods. However, it is important to consider that in human TLE patents sclerosis is the most common cell loss pattern; therefore, alterations in animals with similar cell loss are likely to be relevant in human TLE.

The death of CB1 KO animals with strong acute seizures suggest that CB1 receptors may have a key role in the control of the first seizures, and thereby may prevent the seizures from reaching the “no-return” state. Thus, the decrease of CB1-Rs in animals with strong acute seizures may lead to elevation of glutamate release during acute seizures, as well as to the subsequent development of recurrent seizures, reorganization and cell loss. The increased density of CB1-Rs in the chronic phase may serve as a protective mechanism in most cases as proposed in earlier studies, and confirmed in the present study as well [26], [52].
